# Infectious Pepper Mild Mottle Virus and Human Adenoviruses as Viral Indices in Sewage and Water Samples

**DOI:** 10.1007/s12560-022-09525-0

**Published:** 2022-06-17

**Authors:** Mohammed Kamal Rashed, Waled Morsy El-Senousy, ElSayed Tarek Abd ElSalam Sayed, Maha AlKhazindar

**Affiliations:** 1 Environmental Virology Lab, Water Pollution Research Department, Environmental and Climate Change Research Institute and Food-Borne Viruses Group, Centre of Excellence for Advanced Sciences, National Research Centre (NRC), 33 El-Buhouth Street, P. O. 12622, Dokki, Giza, Egypt; 2 Botany and Microbiology Department, Faculty of Science, Cairo University, Cairo, Egypt

**Keywords:** Adenoviruses, Polyomaviruses, Pepper mild mottle virus, Sewage, Water

## Abstract

The objective of this study was to compare human adenoviruses (HAdVs) genome and infectivity, polyomaviruses (JC and BK) genome (JCPyVs) and (BKPyVs), Pepper Mild Mottle Virus (PMMoV) genome and infectivity, and infectious bacteriophages as viral indices for sewage and water samples. One hundred and forty-four samples were collected from inlets and outlets of water and wastewater treatment plants (WTPs), and WWTPs within Greater Cairo from October 2015 till March 2017. Two methods of viral concentration [Aluminium hydroxide (Al(OH)_3_) precipitation method and adsorption-elution technique followed by organic flocculation method] were compared to determine which of them was the best method to concentrate viruses from sewage and water. Although samples with only one litre volume were concentrated using Al(OH)_3_ precipitation method and the same samples with larger volumes (5–20 L) were concentrated using the adsorption-elution technique followed by the organic flocculation method, a non-significant difference was observed between the efficiency of the two methods in all types of samples except for the drinking water samples. Based on the qualitative prevalence of studied viruses in water and wastewater samples, the number of genome copies and infectious units in the same samples, resistance to treatment processes in water and wastewater treatment plants, higher frequency of both adenoviruses and PMMoV genomes as candidate viral indices in treated sewage and drinking water was observed. The problem of having a viral genome as indices of viral pollution is that it does not express the recent viral pollution because of the longer survivability of the viral genome than the infectious units in water and wastewater. Both infectious adenovirus and infectious phiX174 bacteriophage virus showed similar efficiencies as indices for viral pollution in drinking water and treated sewage samples. On the other hand, qualitative detection of infectious PMMoV failed to express efficiently the presence/absence of infectious enteric viruses in drinking water samples. Infectious adenoviruses and infectious bacteriophage phiX174 virus may be better candidates than adenoviruses genome, polyomaviruses genome, and PMMoV genome and infectivity as viral indices for water and wastewater.

## Introduction

Waterborne pathogens transmit diseases to around 250 million people each year resulting in 10–20 million deaths around the globe (Wilkes et al., 2009). The assessment of the microbiological quality of drinking water aspires to protect consumers from illnesses due to the consumption of water that may contain pathogens such as bacteria, viruses, and protozoa, thereby thwarting water-related illness outbreaks. An indicator of microbial water quality is generally one specific species or group of microorganisms, which must have entered the water system at the same time as feces, but this indicator is easier to measure than the full range of microorganisms that pose the health risk (WHO, 1997). Many studies (Liang et al., 2006; Hewitt et al., 2007; Maunula et al., 2009) have associated the outbreaks of waterborne gastroenteritis with a diversity of enteric bacteria and viruses, although recreational exposure to polluted water has often been more linked to viral infections (Vantarakis & Papapetropoulou, 1999). Coliform bacteria, E.coli, and coliphages are normally used as indicators of water quality. However, the presence of the above-mentioned indicators does not always suggest the presence of human enteric viruses. It is important to study human enteric viruses in water. Human enteric viruses can tolerate fluctuating environmental conditions and survive in the environment for long periods becoming causal agents of diarrhoeal diseases. Therefore, the potential of human pathogenic viruses as significant indicators of water quality is emerging. A good indicator should fulfill the following requirements: (1) should be associated with the source of the pathogen and should be absent in unpolluted areas, (2) should occur in greater numbers than the pathogen, (3) should not multiply out of the host, (4) should be at least equally resistant to natural and artificial inactivation as the viral pathogen, (5) should be detectable using easy, rapid and inexpensive procedures, and (6) should not be pathogenic (Bosch, 1998). Human adenoviruses and other viruses have been proposed as suitable indices for the effective identification of such organisms of human origin contaminating water systems (Lin & Ganesh, 2013).

Based on the bacterial indicator, sometimes the judgment about the validity of water for drinking or irrigation is wrong. So, viruses must be examined in treated effluents or drinking water samples to have a correct judgment on the validity of water for drinking or irrigation. But there will be a problem of how to examine more than 100 viruses that may be present in water samples. It will be very fatigued, expensive, and need a very long time. So, the logical alternative is to have a viral index to express the presence/absence of viruses in water samples. This index must have all the conditions of any indicator except for the condition that indicates that it should not be pathogenic because it is a virus itself and this is necessary to express the viral pollution of water as a viral index.

HAdVs have been shown to frequently occur in raw water sources, treated drinking water supplies, urban rivers, and polluted coastal waters and throughout the year (Puig et al., 1994; Tani et al., 1995; Pina et al., 1998; Jiang et al., 2001; Flomenberg, 2005; Bofill-Mas et al., 2006; Dongdem et al., 2009; Jurzik et al., 2010; El-Senousy, Costafreda, et al., 2013) and approximately 90% of the human population is seropositive for one or more serotypes of AdVs (Fong et al., 2010). Besides the conventional polymerase chain reaction (PCR) for detecting AdV genome (Puig et al., 1994), conventional real-time quantitative PCR (qPCR) methods for detecting and quantifying the HAdV (dsDNA) genome are already established (Rames et al., 2016; Bibby et al., 2019), enabling validation of novel testing methods. Infectious HAdVs could be detected and quantified using several methods such as cell culture-polymerase chain reaction (CC-PCR) (El-Senousy et al., 2013b; El-Senousy and Abou El-ela, 2017), integrated cell culture – preceded by reverse transcriptase and qPCR (ICC-RT-qPCR) to quantify mRNA of HAdV (Fongaro et al., 2013), and fluorescence-activated cell sorting assay (Li et al., 2010). Detection of HAdVs infectious units has advantages in comparison with detection of HAdVs genome using PCR. Human infectious viruses which could be determined using primers specific to human strains express the recent contamination of different water types with human viruses because HAdV genome persists longer in water than HAdV infectious units (Donia et al., 2010; El-Senousy et al., 2014; Prevost et al., 2016).

Five human PyV (BKV, JCV, KIV, WUV, and MCV) have been identified (Kean et al., 2009). These viruses are known for producing lifelong, asymptomatic viremia in immunocompetent individuals (Polo et al., 2004). Over 70% of adults harbor antibodies to BKV or JCV HPyVs (Meng & Gerba, 1996; Lukasik et al., 2000). The obligate host specificity and abundance of BKV and JCV in municipal sewage have led to the successful use of these viruses to indicate human fecal pollution in environmental water samples (Albinana-Gimenez et al., 2006; McQuaig et al., 2006; Brownell et al., 2007). Bofill-Mas et al. (2000) suggested that JCV would be a useful indicator of human sewage in the water. The obligate host specificity of viruses such as HPyVs is advantageous for the specific identification of human sources. JCV or BKV have been detected using conventional PCR in raw sewage from all over the globe (Bofill-Mas et al., 2000; McQuaig et al., 2009; Kokkinos et al., 2011). HPyVs are a good candidate since they are routinely found in environmental water samples from different geographical areas with relatively high abundance. HPyVs are highly human-specific, having been detected in human waste from all age ranges and undetected in animal waste samples. Besides, HPyVs show a certain degree of resistance to high temperature, chlorine, UV, and low pH, with molecular signals (i.e., DNA) persisting in water for several months. Recently, various concentration methods (electronegative/positive filtration, ultrafiltration, skim-milk flocculation) and detection methods (immunofluorescence assay, cell culture, PCR, integrated cell culture PCR (ICC-PCR), and qPCR) have been developed and demonstrated for HPyV, which has enabled the identification and quantification of HPyV in various environmental samples, such as sewage, surface water, seawater, drinking water, and shellfish (Rachmadi et al., 2016; Farkas et al., 2020).

Identification of PMMoV in feces was first achieved through viral metagenomics (Zhang et al., 2006). They reported that the most abundant RNA virus in three fecal samples from healthy adults in the USA was PMMoV, comprising 75.7–99.4% of all sequences identified in the fecal RNA viral community. Phylogenetic analysis of PMMoV strains identified in the fecal samples indicated that the PMMoV strains were very different even in two fecal samples collected from the same individual, which implies that the PMMoV circulation in human’s populations is dynamic (Zhang et al., 2006). PMMoV was subsequently detected in feces by regular RT-PCR or RT-qPCR in six (67%) of nine samples in the USA, six (67%) of nine samples in Singapore (Zhang et al., 2006), 19 (95%) of 20 samples in Germany (Hamza et al., 2011), and in the specimens of one (0.48%) of 208 hospitalized children and 22 (7.2%) of 304 adult patients in France (Colson et al., 2010). Although the detection rate varies between studies, likely due to differences in detection methods or exposure to PMMoV (Colson et al., 2010), these studies have demonstrated that the presence of PMMoV in feces is geographically widespread. PMMoV concentrations in feces are high, ranging from 10^5^ to 10^10^ copies/g-feces (dry weight) (Zhang et al., 2006). PMMoV can be found with greater frequency in healthy human feces than pathogenic viruses (Rosario et al., 2009). Strains isolated in human feces are genetically diverse with dynamic fecal populations within an individual and notably remain viable and infectious to host plants (Zhang et al., 2006). One previous study documented interactions of PMMoV with the human immune system and suggested that the virus may cause clinical symptoms in humans, such as fever, abdominal pains, and pruritus; however, these symptoms may have been confounded by spicy food rich in peppers or pepper-based products (Colson et al., 2010). PMMoV has not been detected in fecal samples or intestinal homogenates of most animals, such as turkeys, horses, coyotes, raccoons, sheep, ducks, pigs, and dogs (Rosario et al., 2009; Hamza et al., 2011). Although fecal samples from cows, geese, seagulls, and chickens were sometimes positive for PMMoV, virus concentrations in these samples were much lower (3–4 log_10_) than those in human feces; the originating source of PMMoV in these animals is unclear (Rosario et al., 2009; Hamza et al., 2011). The objective of this study was to compare human adenoviruses genome and infectivity, polyomaviruses (JC and BK) genome (JC-Py-Vs) and (BK-Py-Vs), and Pepper Mild Mottle Virus (PMMoV) genome and infectivity as viral indices for sewage and water samples.

## Materials and Methods

### Sewage and Water Samples

One hundred and forty-four samples were collected from inlets and outlets of water and wastewater treatment plants (WTPs), and WWTPs within Greater Cairo. Forty-eight raw sewage samples were collected from October 2015 till September 2017 (24 from El-Gabal El-Asfar WWTP and 24 from Zenin WWTP), 48 treated effluents of the same raw sewage samples, 24 raw Nile water samples of El-Giza WTP, and 24 drinking water samples of the same WTP. The samples were collected monthly. El-Gabal El-Asfar and Zenin WWTPs use an activated sludge as a treatment technology and the flow rate in El-Gabal El-Asfar was 1,700,000 cubic meters per day (m^3^/day) while the flow rate in Zenin was 330,000 m^3^/day. El-Gabal El-Asfar receives raw sewage from a large area in Cairo Governorate and Zenin receives raw sewage from a large area in El-Giza Governorate.

### Concentration of Viruses Using the Adsorption-Elution Technique

Sewage samples (5 L of raw sewage and treated effluents) and water samples (10 L of Nile water and 20 L of drinking water) were concentrated by filtration through negatively charged nitrocellulose membranes (ALBET-Spain, 0.45 µm pore size, and 142 mm diameter filter series) after addition of AlCl_3_ to a final concentration of 0.5 mM and acidification to pH 3.5 and after passing through Whatmann No. 1 filter paper. The viruses adsorbed to the membrane were eluted with 75 ml of 0.05 M glycine buffer, pH 9.5 (using HCl 5 N) containing 3% beef extract (Lab-Limco powder, OXOID, UK) (Smith & Gerba, 1982; Rose et al., 1984). All samples were reconcentrated using an organic flocculation method (Katzenelson et al., 1976). Briefly and according to them, the eluate was acidified to pH 3.5 using HCl (5 N) and centrifuged at 3000 rpm for 15 min, the supernatant was discarded, and the pellet was dissolved in 1 ml of Na_2_HPO_4_ (0.14 N, pH 9). The pH of the solution was neutralized by adding 0.1 N HCL and the samples were kept at -70 C until used.

### Concentration of Viruses Using (Al(OH)_3_) Precipitation Method

Sewage and water samples (1 L of each sample) were concentrated using (Al(OH)_3_) precipitation method according to Standard Methods for the Examination of Water and Wastewater (APHA, 2017).

### Viral Nucleic Acid Extraction

Viral RNA was extracted from 140 µl of the supernatant using BIOZOL Total RNA Extraction reagent (BIOFLUX—Japan) and according to the manufacturer’s instructions and a 30 µl final volume was obtained.

### Extraction of DNA

It was done as described previously by Kapperud et al. (1993) and modified by Estrada et al. (2007). 50µL of sample concentrate were added to 50 µl of 1X PCR buffer containing 0.2 mg of Proteinase K/mL. After being incubated at 37 °C for 1 h, the suspension was boiled for 10 min and then centrifuged at 12,500 rpm for 5 min at 4 °C. The supernatant was used for performing the PCR.

### Detection of Adenoviruses Using Nested PCR

It was done according to Puig et al., (1994) using the specific primers hex AA 1885, hex AA 1913 for the first round PCR, and nehex AA 1893 and nehex AA 1905 for the second round PCR for detection of human adenovirus and were selected from the DNA sequence of the open reading frame of hexon gene. PCR products (10 µl) were analyzed by electrophoresis on 3% agarose gels (Panreac-spain).

### Real-Time PCR for Quantification of Adenoviruses

Real-time TaqMan PCR was performed for positive samples in the previous PCR screening. Real-time PCR was done using adenovirus@ceeramTools™ Food & Environmental kit and according to the manufacturer’s instructions using adenovirus—Q Standard (Ceeram Tools) and Mengo Extraction Control (Ceeram Tools) and using a real-time PCR thermal cycler (Rotor-Gene Q, Qiagen). Raw adenovirus genome copies numbers measured by real-time RT-PCR, in duplicate, were corrected according to virus/nucleic acid extraction and RT-PCR efficiencies.

### Cell Culture-PCR (CC-PCR) Technique for Quantification of Adenovirus Infectious Units

It was done according to Esawy et al. (2011) and Abdo et al. (2012) Adenovirus cell culture-PCR (CC-PCR) assay was performed on suspensions of the infected Hep-2 cell line. A set of primers, hex AA 1885 and hex AA 1913, was used. The detection limit in this tissue culture assay using 100 μl of inoculum is 1 × 101 CC-PCR units/ml (u/ml). An adenovirus CC-PCR unit is defined as the reciprocal endpoint dilution detectable by CC-PCR.

### PCR for Detection of Polyomaviruses JC and BK

It was performed according to Bofill-Mas et al. (2000). Amplification was carried out in a 50 μl reaction mixture containing 10 mM Tris–HCl (pH 8.3 at 25 °C), 50 mM KCl, 1.5 mM MgCl2, 200 μM (each) deoxynucleoside triphosphate, 2 U of Ampli Taq DNA polymerase (Thermo-Fisher), and the corresponding primers at their corresponding concentrations (0.5 μM external and internal primers for all polyomavirus amplifications). Thermal cycling of the amplification mixture was performed in a programmable heat block (Applied Biosystem). In all PCR assays for polyomavirus detection, the first cycle of denaturation was carried out for 4 min at 94 °C. The conditions for the 29-cycle amplification were as follows: denaturing at 92 °C for 60 s, annealing for 60 s, and extension at 72 °C for 75 s. Amplifications were completed with a 4 min, 72 °C extension period. JCV genomes were amplified using EP1A 5′-TGAATGTTGGGTTCCTGATCCCACC-3′ and EP2A 5′-ACCCATTCTTGACTTTCCTAGAGAG-3′ as external primers and P1A 5′-CAAGATATTTTGGGACACTAACAGG-3′ and P2A 5′-CCATGTCCAGAGTCTTCTGCTTCAG-3′ as internal primers and an annealing temperature of 59 °C in both PCRs. BKV genomes were amplified using external primers BK1 5′-TATTGCCCCAGGAGGT-3′ and BK2 5′-AACATTTTCCCCTCCTG-3′ at an annealing temperature of 46 °C and internal primers BK4 5′-AGTAGATTTCCACAGGTTAG-3′ and BK6 5′-CCAGGGGCAGCTCCCAAAAAG-3′ at an annealing temperature of 50 °C. Positive samples were confirmed using DNA sequencing.

### Mechanical Inoculation of PMMoV to Examine Viral Infectivity in the Plant Host

Two months-old Capsicum annuum L. plants (common name pepper) were used to test the infectivity of PMMoV in the water and sewage samples. Three plants were used as replicates for each concentrated sample using either (Al(OH)_3_) precipitation method or adsorption-elution technique followed by organic flocculation method of the 144 treated and untreated sewage and water samples (the Al(OH)3 method and adsorption-elution technique followed by organic flocculation method besides the non-concentrated samples). Each sample was applied to the leaves (50 Ul/ leaf) after dusting the leaves with 600-mesh Carborandum. The leaf was supported in the palm of the left hand with the leaf apex pointing towards the wrist and the petiole downward between the second and the third fingers. The leaves were rubbed firmly but gently over the entire upper surface and then washed immediately with tap water (Walkey, 1991). Control pepper samples were inoculated with double distilled water using the same technique. Plants were kept in temperature-controlled (20–25 °C), insect-proof greenhouse and monitored.

### RT-PCR for Detection of PMMoV

It was performed according to Zhang et al., (2006). The pairs of specific primers 5′- AACCTTTCCAGCACTGCG-3′ (forward) and 5′-GCGCCTATGTCGTCAAGACT-3′ (reverse) were used in RT-PCR for the amplification of the replication-associated protein (201 bp). Positive samples were confirmed using DNA sequencing. This RT-PCR technique was performed either to analyze concentrated samples or to confirm the specificity of the positive samples after inoculation in pepper plants to show the infectivity of PMMoV.

### Confirmation of the Positivity of Polyomaviruses and PMMoV by Amplimer Sequencing

The PCR products of positive samples for polyomaviruses and PMMoV were sequenced. Fifty to one hundred µl of the PCR products were purified using a high pure PCR products purification kit (Qiagen) following the manufacturer’s instructions. Sequencing was performed on 1–7 µl of the purified products with an ABI prism Big dye termination cycle sequencing ready reaction kit (Applied Biosystem) using the same primers as in the PCR and following the manufacturer’s instructions. The DNA was sequenced with an ABI prism 310 automated DNA sequencer. Sequence data from both strands of the PCR products were aligned and compared using the CLUSTALW and BLAST programs (European Bioinformatics Institute).

### Quantification of Infectious Bacteriophage phi X174 Virus

It was performed according to the standard methods for the examination of water and wastewater, 23rd edition (APHA, 2017). Bacteriophage phiX174 strain (ATCC 13706B1) and Escherichia coli strain C (ATCC 13,706) ATCC.

### Statistical Methods

A paired Student's t test was applied to ascertain the significance at *p* < 0.05 of differences of virus recovery after (Al(OH)_3_) precipitation method and adsorption-elution technique followed by organic flocculation method and on the other hand, the differences of virus recovery between concentrated and non-concentrated samples.

## Results

**Fig. 1:**
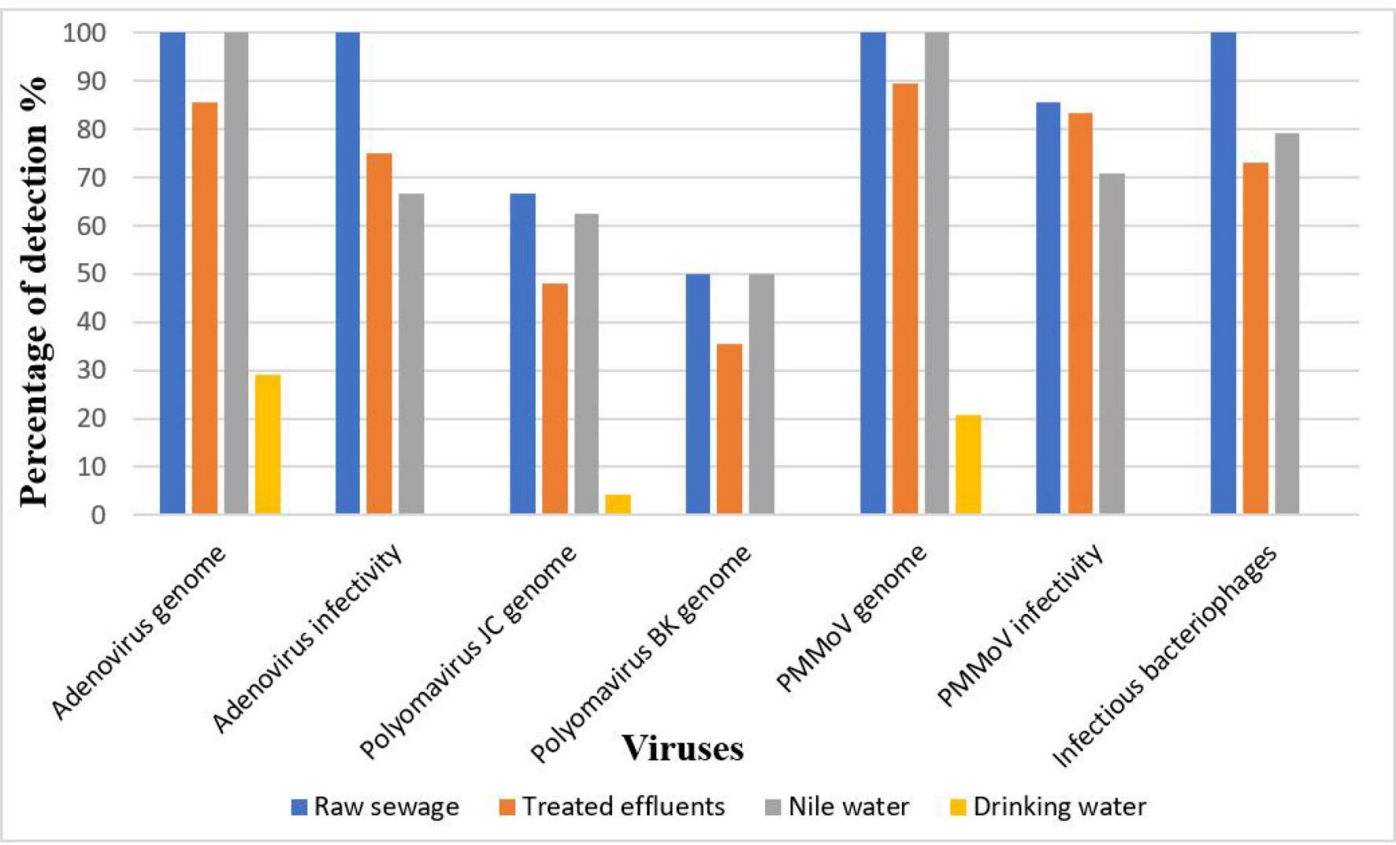
Frequency of adenoviruses, Polyomavirus JC, Polyomavirus BK, and PMMoV in different water types using Al (OH)_3_ precipitation method

**Fig. 2:**
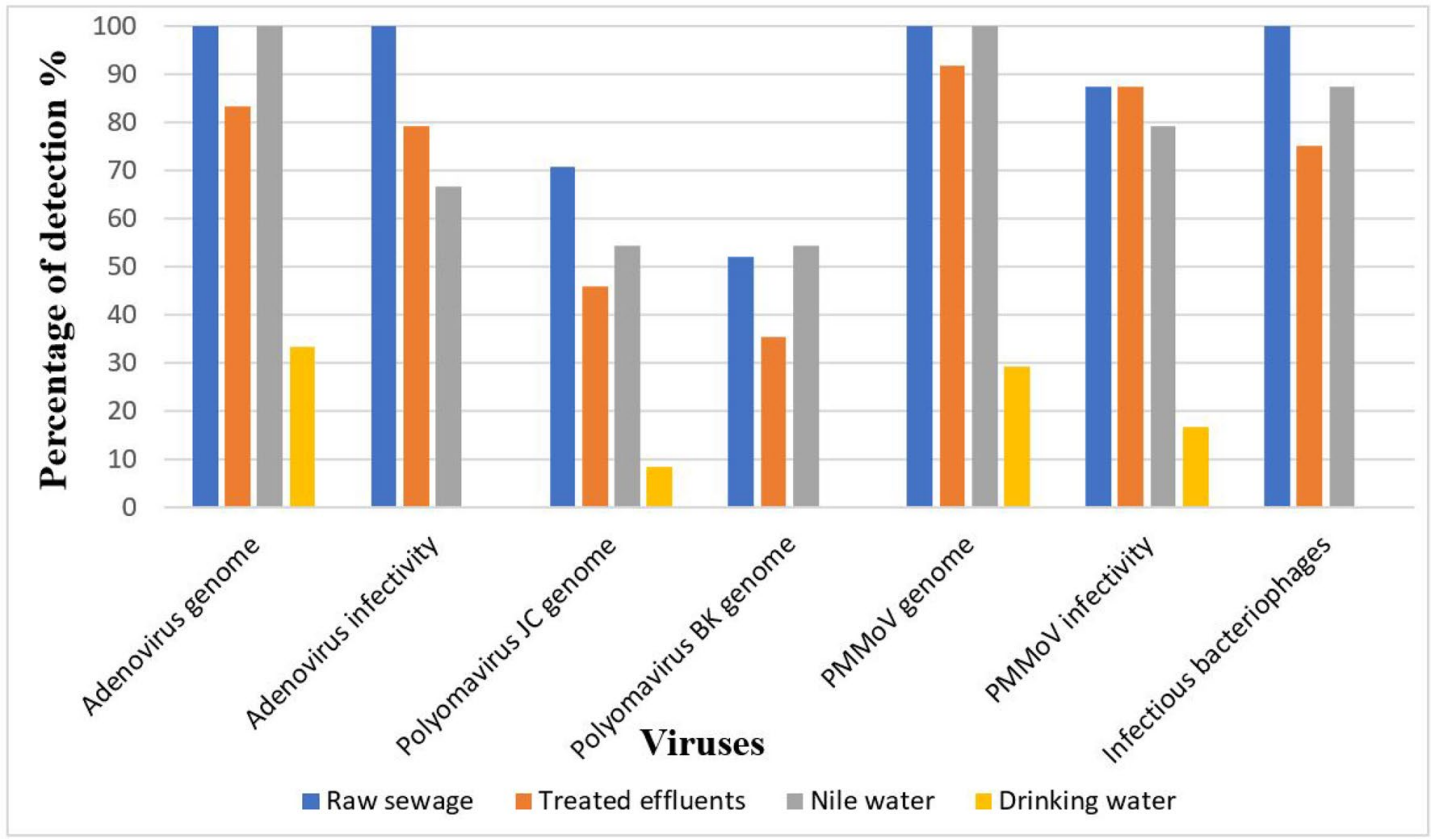
Frequency of adenoviruses, Polyomavirus JC, Polyomavirus BK, and PMMoV in different water types using adsorption-elution/organic flocculation technique

The results of prevalence of adenovirus genome and infectious units, polyomavirus JC genome, polyomavirus BK genome, PMMoV genome and infectivity, and infectious units of phiX174 bacteriophage in raw sewage, treated effluents, Nile water, and drinking water concentrated by both Al(OH)_3_ precipitation method and adsorption-elution/organic flocculation technique are shown in Figs. 1 and 2. Using both concentration methods, the frequency of both adenovirus and PMMoV genome was higher than the frequency of both polyomavirus JC and polyomavirus BK genomes in raw sewage and Nile water samples. No significant difference in the frequency of genome copies of adenovirus, polyomavirus, and PMMoV and infectious units of adenovirus, PMMoV, and phiX174 bacteriophage virus using the two concentration methods was observed in all types of samples except for the drinking water samples, where adsorption-elution/organic flocculation showed higher sensitivity.

**Fig. 3:**
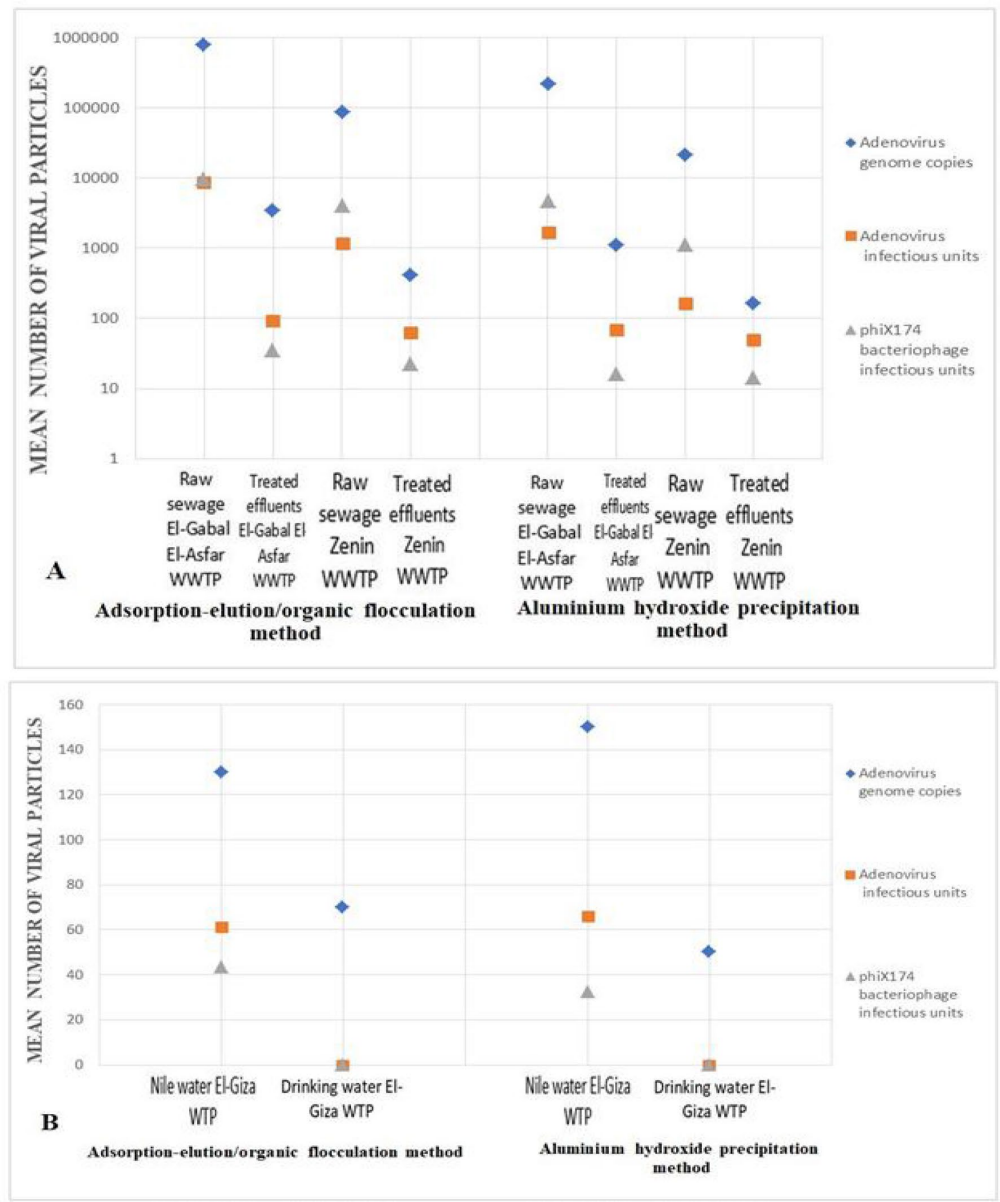
Mean number of Genome copies and infectious units of adenoviruses and infectious units of phiX174 bacteriophage in sewage and water samples using both adsorption-elution/organic flocculation and aluminium hydroxide precipitation methods. **A** sewage samples (raw and treated), **B** water samples (Nile water and drinking water)

The results of the mean numbers of adenovirus genome and infectious units and infectious units of phiX174 bacteriophage in raw sewage, treated effluents, Nile water, and drinking water concentrated by both Al (OH)_3_ precipitation technique and adsorption-elution/organic flocculation technique are shown in Fig. 3. No significant difference in the number of viral genome copies of adenovirus and infectious units of adenovirus and phiX174 bacteriophage virus using the two concentration methods was observed in all types of samples.

Results of the number of infectious particles (range and median) of adenovirus, PMMoV, and phiX174 bacteriophage virus in the non-concentrated raw sewage, treated effluents, Nile water, and drinking water samples are shown in Table 1. Using direct inoculation from non-concentrated samples, the phiX174 bacteriophage virus is the only virus detected in all types of samples except for the drinking water samples.

**Table 1:** Range and median values of infectious particles of adenovirus, PMMoV, and phiX174 bacteriophage virus in the non-concentrated sewage and water samples

Viruses type of samples	Adenovirus genome copies/litre	Adenovirus infectious units/litre	phiX174 bacteriophage infectious units/litre	PMMoV genome	PMMoV infectivity
Raw sewage samples El-Gabal Al-Asfar WWTP	0	0	9 × 10^3^ − 2 × 10^5^ (4.8 × 10^4^)	0	0
Raw sewage samples Zenin WWTP	0	0	5 × 10^3^ − 1.5 × 10^5^ (3.2 × 10^4^)	0	0
Treated effluents El-Gabal Al-Asfar WWTP	0	0	0 − 4.4 × 10^3^ (4.7 × 10^2^)	0	0
Treated effluents El-Zenin WWTP	0	0	0 − 1.2 × 10^3^ (6.5 × 10^2^)	0	0
Nile water El-Giza WTP	0	0	0 − 1.3 × 10^2^ (1.5 × 10^2^)	0	0
Drinking water of El-Giza WTP	0	0	0	0	0

**Fig. 4:**
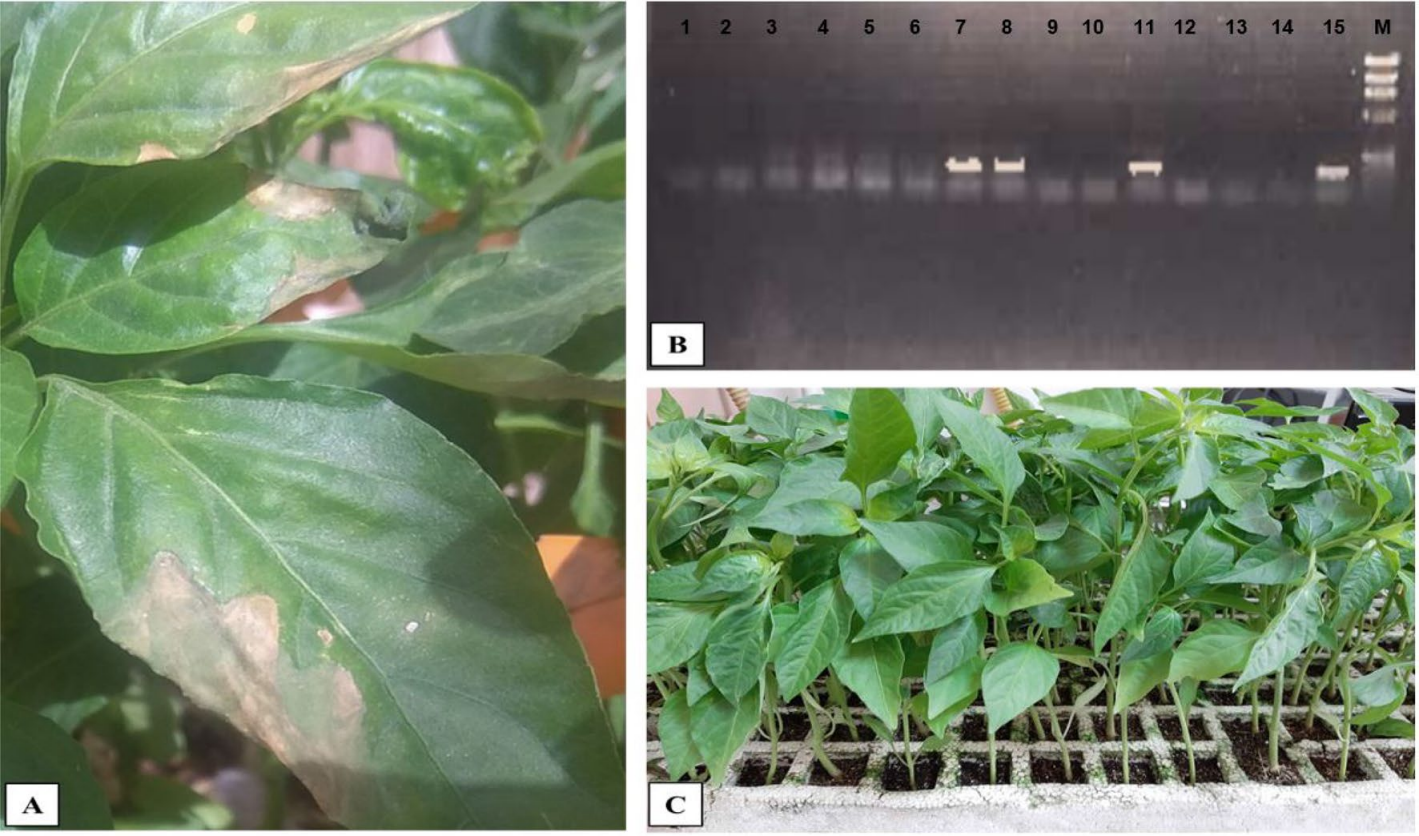
Effect of PMMoV on pepper plant leaves. **A:** Symptoms of PMMoV on pepper leaves after 1 week of inoculation of pepper plant with raw sewage sample **B:** RT-PCR results for confirmation of specificity of PMMoV in causing these symptoms (positive sewage and water samples lanes 7, 8, and 11 showed 201 bp bands, and lane 15 positive control from RNA of positive sample which previously was analyzed and negative control lane 1, while negative sewage and water samples lanes 2, 3, 4, 5, 6, 9, 10, 12, 13, and 14). **C:** Control pepper plant which was not inoculated with PMMoV after the same period (1 week)

Specificity of symptoms appeared on the leaves of pepper plants after inoculation of concentrated and non-concentrated sewage and water samples were confirmed in all positive samples (100%) using RT-PCR/sequencing (Fig. 4). All samples taken from the lesions which appeared on the surface of the pepper plants showed 201 bp fragments which were confirmed by sequencing.

## Discussion

The objective of this study was to compare human adenoviruses genome and infectivity, polyomaviruses (JC and BK) genome (JCPyVs) and (BKPyVs), Pepper Mild Mottle Virus (PMMoV) genome and infectivity, and infectious bacteriophages as viral indices for sewage and water samples. To achieve this objective, adenovirus genome and infectious units, JCPyVs genome, BKPyVs genome, PMMoV genome, PMMoV infectivity, and infectious bacteriophages were investigated in sewage and water samples collected monthly from two WWTPs and one WTP inside Greater Cairo. The concentration of samples using (Al(OH)_3_ precipitation method and adsorption-elution technique followed by an organic flocculation method did not show a significant difference in the efficiency in raw sewage, treated effluent, and raw Nile water samples. However, a significant difference in the efficiency was observed in drinking water samples. PMMoV infectivity was detected in 16.67% of the drinking water samples concentrated using the adsorption-elution technique followed by an organic flocculation method, while no infectivity was detected in the same samples concentrated using the (Al(OH)_3_ precipitation method. The difference of samples, volume may be the reason for the superiority of the adsorption-elution technique which twenty litres of the drinking water samples were concentrated, however, only one litre was concentrated using the (Al(OH)_3_ precipitation method. This difference in samples, volume was not effective with the other types of samples. This may return to the higher viral concentration in raw sewage, treated effluent, and raw Nile water samples than its concentration in the drinking water samples. Another reason is that there is no big difference between the volumes of the samples concentrated using the two methods in these types of water. A lot of previous studies showed higher viral frequency in treated and untreated sewage and river water than its frequency in the drinking water samples (Keswick et al., 1984; Gilgen et al., 1995; Reynolds et al., 1997; Fong & Lipp, 2005; da Silva et al., 2007; Arraj et al., 2008; Rodríguez et al., 2012; El-Senousy et al., 2013; Betancourt et al., 2014; El-Senousy et al., 2015; Kitajima et al., 2018). This may lead us to use (Al(OH)_3_ precipitation method with smaller volumes with these types of samples; however this concentration method is not suitable for the viral concentration from the drinking water samples. Increasing the samples, volume may improve the efficiency of the (Al(OH)_3_ precipitation method to concentrate viruses from drinking water samples.

In this study, genomes of both adenoviruses and PMMoV have a higher frequency than the genomes of JCPyVs and BKPyVs in raw sewage and raw Nile water. Also, they have a higher resistance rate in both treated sewage and drinking water. Higher frequency of both adenoviruses and PMMoV was previously reported (Rosario et al., 2009; Hamza et al., 2011; Wong et al., 2012; Haramoto et al., 2013; Kitajima et al., 2014; Kuroda et al., 2015; Hughes et al., 2017). Also, higher resistance to water and wastewater treatment processes and especially chlorine disinfection was previously reported (Rosario et al., 2009; Hamza et al., 2011; Kuroda et al., 2015; Schmitz et al., 2016; Hughes et al., 2017; Symonds et al., 2018). Although, this may give a tendency to adenoviruses and PMMoV genomes as candidate viral indices in treated sewage and drinking water, the problem of having viral genome as an index of viral pollution is that it does not express the recent viral pollution because of the longer survivability of viral genome than the infectious units in water and wastewater (Donia et al., 2010; El-Senousy et al., 2014; Prevost et al., 2016; El-Senousy, 2021). Although, our results showed comparable frequency for infectious units of adenoviruses, PMMoV, and phiX174 bacteriophage viruses in raw sewage and raw Nile water, higher resistance of infectious units of PMMoV to water and wastewater treatment processes than adenoviruses and phiX174 bacteriophage virus was observed in treated sewage and drinking water. This may return to the higher resistance of PMMoV to chlorine disinfection than adenoviruses (Kitajima et al., 2018; Shirasaki et al., 2018, 2020) and bacteriophages (Shirasaki et al., 2018). This was clear in the appearance of PMMoV infectivity four times in drinking water samples, while the complete absence of the infectious units of both adenoviruses and phiX174 bacteriophage virus was observed in these samples. This may indicate the failure of PMMoV infectious units to express the pollution of treated sewage and drinking water with enteric viruses effectively when it was detected qualitatively. These results were supported by Shirasaki et al., (2018) who suggested that PMMoV is not useful as a surrogate for enteric viruses concerning free-chlorine disinfection processes. More research is needed to examine the efficiency of quantitative assays of infectious PMMoV to express the enteric viruses, pollution of treated sewage, and drinking water.

In this study, quantification assays of infectious units of adenoviruses and phiX174 bacteriophage virus showed closely related results in raw sewage, raw Nile water, treated sewage, and drinking water samples using the two concentration methods. This may indicate similar frequency patterns in raw sewage and water for both viruses and similar resistance capability to water and wastewater treatment processes. On the other hand, when sewage and water samples were inoculated directly without a concentration process, a significant difference between the numbers of both viruses in all water types was observed. This may return to the lower efficiency of both concentration methods for concentrating the bacteriophage phiX174 in comparison to adenoviruses. Another possibility is the higher viral load of bacteriophage phiX174 virus in raw sewage, treated effluents, and raw Nile water samples than adenoviruses and because of the lower efficiency of the concentration methods, the number of both viruses looked convergent after both concentration methods.

This is the first time to examine the infectivity of PMMoV by inoculation of concentrated and non-concentrated samples of treated and untreated sewage and water in the leaves of pepper plants to detect the infectious PMMoV in these types of water. Zhang et al., (2006) inoculated stool specimens in the leaves of pepper plants to show the viral infectivity through the lesions that appeared on the surfaces of the leaves. The lesions which were appeared on the surfaces of some plant leaves in our study had a similar appearance in all of them. Specificity of the causative agents was confirmed using RT-PCR followed by sequencing and all of the samples taken from the different lesions showed positive results as PMMoV. The integrated method using inoculation of the pepper leaves followed by RT-PCR/sequencing for the samples taken from the lesions on some leaves is a necessary method till now to confirm the specificity of the test.

Conclusions may be summarized as follows: first, infectious adenoviruses and infectious bacteriophage phiX174 virus may be better candidates than adenoviruses genome, polyomaviruses genome, and PMMoV genome and infectivity as viral indices for water and wastewater. However, both (Al(OH)_3_) precipitation method and adsorption-elution technique on nitrocellulose membranes followed by organic flocculation method have lower efficiency when concentrating bacteriophage phiX174 than their efficiencies when concentrating adenoviruses. This may give an advantage for the infectious adenoviruses as a viral index in water and wastewater. Second, both (Al(OH)_3_) precipitation method and adsorption-elution technique on nitrocellulose membranes followed by organic flocculation method have comparable efficiencies as viral concentration methods from raw sewage, treated effluents, and raw Nile water (river water), while, adsorption-elution technique on nitrocellulose membranes followed by organic flocculation method is better to be used with drinking water samples. Third, an integrated method that contains inoculation of concentrated water and wastewater samples in the leaves of pepper plants followed by RT-PCR is necessary to confirm the specificity of the causative agent of the appeared lesions as PMMoV.
